# Speed-related activation in the mesolimbic dopamine system during the observation of driver-view videos

**DOI:** 10.1038/s41598-017-18792-y

**Published:** 2018-01-15

**Authors:** Hiroyuki Sakai, Takafumi Ando, Norihiro Sadato, Yuji Uchiyama

**Affiliations:** 1 Human Science Research Domain, Toyota Central R&D Laboratories, Inc, Nagakute, Aichi 480-1192 Japan; 2 Division of Cerebral Integration, National Institute of Physiological Sciences, Okazaki, Aichi 444-8585 Japan

## Abstract

Despite the ubiquity and importance of speeding offenses, there has been little neuroscience research regarding the propensity for speeding among vehicle drivers. In the current study, as a first attempt, we examined the hypothesis that visual inputs during high-speed driving would activate the mesolimbic dopaminergic system that plays an important role in mediating motivational craving. To this end, we used functional magnetic resonance imaging to identify speed-related activation changes in mesolimbic dopaminergic regions during the observation of driver-view videos in two groups that differed in self-reported speeding propensity. Results revealed, as we expected, greater activation in the ventral tegmental area (VTA) in response to driver-view videos with higher speed. Contrary to our expectation, however, we found no significant between-group difference in speed-related activation changes in mesolimbic dopaminergic regions. Instead, an exploratory psychophysiological interaction analysis found that self-reported speeding propensity was associated with speed-related functional coupling between the VTA and the right intraparietal sulcus. Further validation of our hypothesis will require future studies examining associations between speed-related activation in the mesolimbic dopaminergic system and individual differences in speeding propensity, using a more reliable measure of actual speeding propensity in real traffic.

## Introduction

The propensity for speeding is widespread. To our knowledge, all motorized countries have penal rules for excessive speeding. These rules are typically based on the premise that people often fail to stay within designated speed limits, reflecting the common propensity for speeding among drivers. Interestingly, speed-related vehicle specifications are often emphasised as a major contributing factor of *driving pleasure*, particularly in the context of car advertising^1^. This is also indicative of the prevailing propensity for speeding in the general population.

Despite the ubiquity and importance of speeding offenses, there has been very little neuroscience research regarding speeding propensity. Beeli *et al*.^2^ reported that anodal transcranial direct current stimulation over the dorsolateral prefrontal cortex reduced the number of speeding violations in a simulated driving environment. This suggests that the dorsolateral prefrontal cortex plays a role in risk aversion or legal compliance in car driving situations, but does not provide information about the underlying neural mechanisms of speeding propensity per se. Meanwhile, Erk *et al*.^3^ explored brain activation in response to images of various types of car, revealing that the ventral striatum was selectively activated by images of sports cars. Sports cars are a symbol of high speed, and the ventral striatum is known to be part of the reward system in the brain, constituting a crucial structure for behavioural motivation. Thus, these findings suggest the possibility that car-related visual cue responses in the ventral striatum might be associated with speeding propensity. However, because sports cars are also a cultural icon signalling wealth and social dominance, Erk *et al*.^3^ concluded that the ventral striatum activation they observed in response to images of sports car may be likely to reflect brain responses to cultural properties of objects. Although many studies have identified brain regions associated with different aspects of car driving^4–15^, no previous studies have directly investigated the issue of speeding propensity.

In the current study, as a first attempt to identify the neural substrates underlying speeding propensity, we hypothesised that sensory inputs during high-speed driving would activate the brain reward system. Humans commonly crave sensory inputs that give rise to pleasant sensations, and abundant evidence indicates that the craving for pleasant sensations is associated with activation within the brain reward system^16–22^. Taken together, these findings suggest that the sensory cue reactivity in the brain reward system is critical for craving. To validate our hypothesis, we used functional magnetic imaging (fMRI) to explore activation in response to visual inputs during high-speed driving within mesolimbic dopaminergic regions, the core of the brain reward system. This study had two specific aims: (1) to examine speed-related activation changes in mesolimbic dopaminergic regions of interest (ROIs) during the observation of driver-view videos and (2) to compare speeding-related activation changes between two groups that would be expected to differ in speeding propensity (participants with and without a self-reported interest in sports cars).

## Results

### Group characteristics

**Figure 1:**
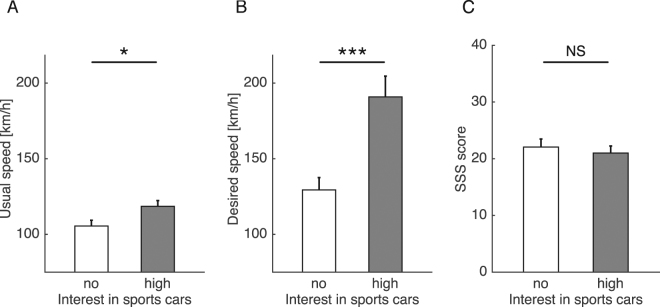
Group characteristics. Participants’ usual speed on actual expressways with a speed limit of 100 km/h (**A**) and participants’ desired speed on expressways with no speed limit (**B**) were compared between participants who had high interest in sports cars and those who had no interest. In addition, to evaluate general propensities for stimulating and arousing experiences, between-group difference in sensation-seeking scale (SSS) score were also compared (**C**). Error bars represent the standard error. Single (*P* < 0.05) and triple (*P* < 0.001) asterisks indicate statistical significance. NS denotes that differences were not significant (*P* > 0.1).

Participants’ self-reported usual speed on expressways with a speed limit of 100 km/h was significantly higher for participants who had high interest in sports cars, compared with those who had no interest (no interest: mean (M) = 105, standard error (SE) = 4 km/h; high interest: M = 119, SE = 4 km/h, *P* = 0.023; Fig. 1A). In addition, there was a significant between-group difference in participants’ desired speed on expressways with no speed limit (no interest: M = 129, SE = 8 km/h; high interest: M = 191, SE = 14 km/h, *P* < 0.001; Fig. 1B). However, the proportion of speeding offenders did not differ between groups (no interest: 3/16; high interest: 4/17, *P* = 0.74). In addition, there was no significant between-group difference in general propensities for stimulating and arousing experiences as assessed by the sensation-seeking scale (SSS) score (no interest: M = 22.1, SE = 1.4; high interest: M = 21.0, SE = 1.2, *P* = 0.58; Fig. 1C). SSS scores were not significantly correlated with usual speed (*r* = 0.077, *P* = 0.67) or desired speed (*r* = 0.055, *P* = 0.76).

### Brain activation

**Figure 2:**
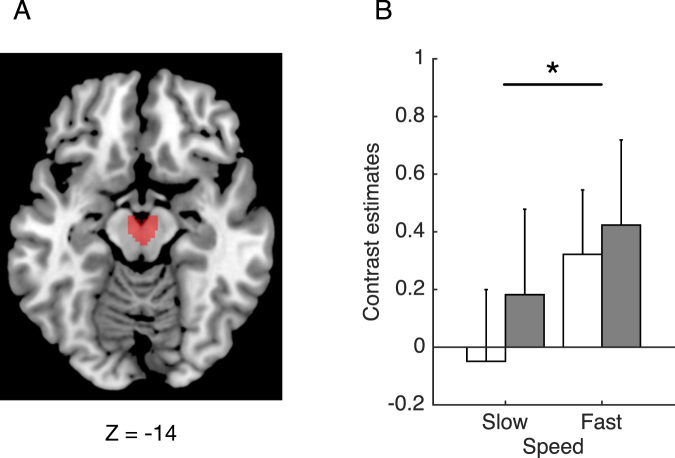
Activation of the ventral tegmental area (VTA) during the observation of driver-view videos. The VTA region of interest (**A**) was anatomically determined in accordance with the midbrain probabilistic atlas provided by Murty *et al*.^48^. Bar graphs depict contrast estimates in the VTA (**B**). White and grey bars represent the mean contrast estimates in participants who had no interest in sports cars and those who had high interest, respectively. Error bars denote the standard error. A single asterisk indicates the statistical significance in the main effect of speed (*P* < 0.05).

**Figure 3:**
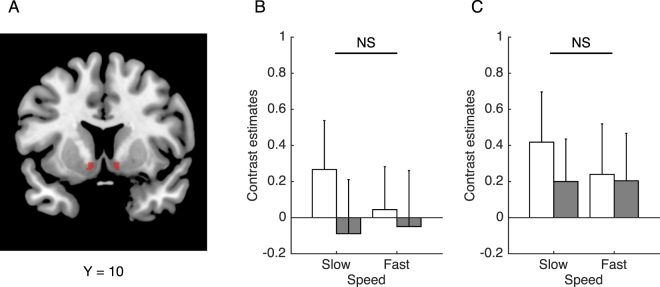
Activation of the nucleus accumbens (NAc) during the observation of driver-view videos. The NAc region of interest was anatomically determined in accordance with the Harvard-Oxford subcortical atlas (**A**). Bar graphs depict contrast estimates in left (**B**) and right (**C**) NAc. White and grey bars represent the mean contrast estimates in participants who had no interest in sports cars and those who had high interest, respectively. Error bars denote the standard error. NS indicates no significance in the main effect of speed (*P* > 0.1).

To examine speed-related activation changes of mesolimbic dopaminergic regions, we compared activation during the observation of driver-view videos recorded from a car traveling in two different speed conditions (slow: M = 41, standard deviation (SD) = 1 km/h; fast: M = 124, SD = 4 km/h) in ROIs for the ventral tegmental area (VTA; Fig. 2A) and the bilateral nucleus accumbens (NAc; Fig. 3A). Figure 2B shows the mean contrast estimates for the VTA ROI. In accord with our hypothesis, the VTA was significantly more active in the fast compared with the slow condition. However, there appeared to be no systematic change in contrast estimates in relation to interest in sports cars. Analysis of variance (ANOVA) revealed a significant main effect of stimulus condition (*F*(1, 31) = 9.29, *P* = 0.0047), but no significant main effect of group (*F*(1, 31) = 0.20, *P* = 0.66) and no significant interaction between group and stimulus condition (*F*(1, 31) = 0.41, *P* = 0.53). Figure 3B,C show the mean contrast estimates for the left and right NAc ROIs, respectively. ANOVA revealed that there was no significant main effect of stimulus condition (left: *F*(1, 31) = 0.26, *P* = 0.61; right: *F*(1, 31) = 0.32, *P* = 0.58), no significant main effect of group (left: *F*(1, 31) = 0.40, *P* = 0.53; right: *F*(1, 31) = 0.14, *P* = 0.71) and no significant interaction between group and stimulus condition (left: *F*(1, 31) = 0.53, *P* = 0.47; right: *F*(1, 31) = 0.35, *P* = 0.56). Further, SSS scores were not significantly correlated with speed-related activation changes (i.e., fast *vs*. slow) in any ROIs (VTA: *r* = 0.21, *P* = 0.23; left NAc: *r* = 0.039, *P* = 0.83; right NAc: *r* = 0.12, *P* = 0.45).

**Figure 4:**
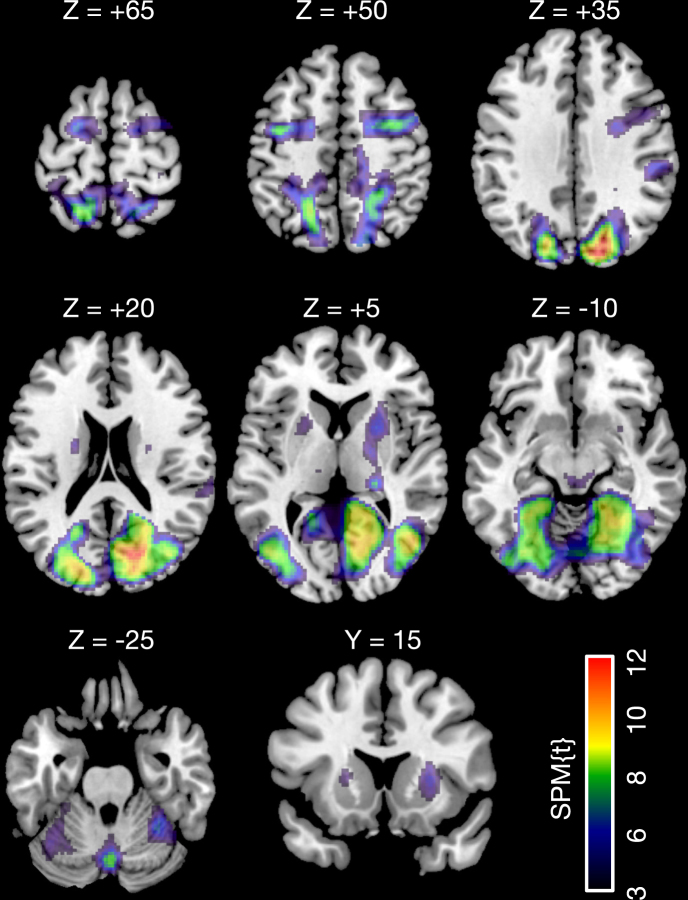
Results of whole brain analysis. Brain regions showing greater activation in the fast compared with the slow condition are rendered on a standard brain template (seven axial slices and surface). The level of statistical significance was set at *P* < 0.05, family-wise error corrected for multiple comparisons at the cluster level with a height-threshold of *P* < 0.001 at the voxel level.

An exploratory whole-brain analysis showed greater activation in the fast compared with the slow condition in extensive cortical and subcortical regions including visual areas in both the dorsal and ventral streams, intraparietal sulcus (IPS), frontal eye field, putamen, thalamus, brain stem and cerebellar vermis (Table 1; Fig. 4). In accord with the ROI analysis, no brain regions showed a significant main effect of group or a significant interaction between group and stimulus condition. In addition, there were no regions in which speed-related activation changes were significantly correlated with SSS scores.

**Table 1:** Brain regions showing greater activation in the fast compared with the slow condition.

Extent	Brain region	Peak coordinates			T-score
		x	y	z	
33,305	R lingual gyus	10	−60	0	11.26
	L superior parietal lobule	−20	−54	58	10.71
	R inferior occipital gyrus	48	−68	4	9.90
	L lingual gyrus	−26	−48	−6	8.63
	Cerebellar vermis	−4	−68	−34	8.60
	L inferior occipital gyrus	−44	−72	6	8.45
	R middle occipital gyrus	40	−74	20	8.16
	L fusiform gyrus	−28	−68	−8	7.48
	R superior parietal lobule	16	−58	62	7.14
	R thalamus	24	−28	6	6.42
	R putamen	24	8	4	5.36
2,333	R middle frontal gyrus	38	0	50	6.37
1,519	L precentral gyrus	−36	−4	48	6.77
575	R supramarginal gyrus	66	−28	22	4.29
322	L putamen	24	6	0	4.17

**Note**. Peak coordinates are given in Montreal Neurological Institute space. The statistical significance level was set to family-wise error corrected *P* < 0.05 at the cluster level with a height-threshold of *P* < 0.001 at the voxel level. L and R denote left and right hemispheres, respectively.

### Psychophysiological interaction (PPI)

**Figure 5:**
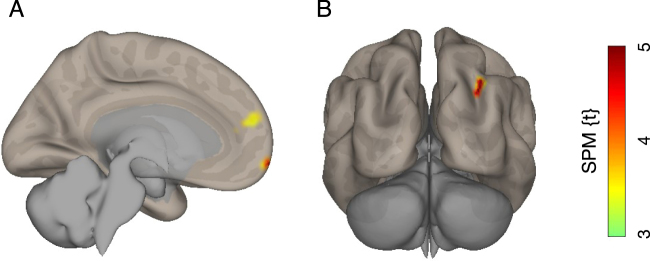
Results of psychophysiological interaction analysis. Brain regions showing reater functional coupling with the VTA in the fast compared with the slow condition are rendered on a standard brain template (**A**). In addition, a brain region showing greater speed-related functional coupling with the VTA in participants with high interest in sports cars, compared with participants with no interest. The level of statistical significance was set at *P* < 0.05, family-wise error corrected for multiple comparisons at the cluster level with a height-threshold of *P* < 0.001 at the voxel level.

We next explored speed-related functional connectivity changes between the mesolimbic dopaminergic ROIs and the rest of the brain. The results confirmed that functional connectivity between the VTA and the rostral-ventral parts of the medial prefrontal cortex (medial PFC) were stronger in the fast condition than in the slow condition (Table 2; Fig. 5A). Additionally, speed-related functional connectivity between the VTA and the right IPS was greater in participants who had high interest in sports cars than in those who had no interest (Table 2; Fig. 5B). No regions were found to have significant speed-related differences in functional connectivity with the NAc in any contrast.

**Table 2:** Brain regions showing greater functional coupling with the VTA in the fast compared with the slow condition.

Extent	Brain region	Peak coordinates			T-score
		x	y	z	
*Across all participants*					
244	Paracingulate gyrus	10	54	18	4.84
133	Frontal pole	48	−68	4	5.00
*High* > *No interest in sports cars*					
113	Right intraparietal sulcus	32	−64	36	5.34

Note. Peak coordinates are given in Montreal Neurological Institute space. The statistical significance level was set to family-wise error corrected *P* < 0.05 at the cluster level with a height-threshold of *P* < 0.001 at the voxel level.

## Discussion

This study was a first attempt to explore the neural substrates underlying speeding propensity. We hypothesised that sensory inputs during high-speed driving would modify activation within the brain reward system. Thus, the present experiment sought to examine speed-related activation changes of mesolimbic dopaminergic regions during the observation of driver-view videos and to compare speed-related activation between two groups with different propensities for speeding. Regarding speed-related activation changes, the VTA exhibited greater activation in response to video clips recorded from a car traveling at a higher speed. Regarding group differences, as we predicted, participants with high interest in sports cars showed a greater propensity for speeding, compared with those who had no interest. However, contrary to our expectation, we found no significant between-group differences in speed-related activation changes within midbrain dopamine regions.

It is widely accepted that the VTA is active in response to a variety of reward-related sensory inputs, and that such dopaminergic activation is often associated with craving for rewards. For example, Filbey *et al*.^16^ reported greater VTA activation in response to favourite alcohol tastes in heavy alcohol drinkers. Bragulat *et al*.^17^ showed that the VTA was more active in response to odours of preferred foods, in contrast with non-appetitive odours, in food-deprived women. Moreover, even exposure to photographic images of craving targets can trigger activation in the VTA, including smoking-related images for nicotine-deprived smokers^18^, images of partners’ faces for romantic lovers^19,20^, and appetising food images for reward-sensitive^21^ and obese^22^ individuals. The current results further showed that visual input during high-speed driving also trigger VTA activation, potentially mediating a motivational propensity for speeding. This notion is consistent with an animal study reporting that visual stimuli containing rich global motion signals (i.e., action movies) are a more effective reinforcer of operant reactions, compared with still images^23^.

The absence of speed-related activation in the NAc may provide further insight into the interpretation of speed-related VTA activation. The VTA is known to be activated not only by reward relevance of stimuli but also by stimulus novelty. Krebs *et al*.^24^ investigated functional segregation within the VTA using high-resolution fMRI, and found two distinct subregions that exhibited different response characteristics in terms of reward relevance and novelty of stimuli. While one was sensitive to reward relevance rather than novelty, just as it was for the NAc, the other was sensitive to novelty rather than reward relevance. Such a novelty-sensitive response was also found in part of the ventral stream of the visual system, including the fusiform gyrus and the parahippocampal area. In the current study, ROI and whole-brain analyses revealed that the VTA was co-activated with the ventral visual stream regions but not with the NAc. From a computational point of view, novelty-induced dopaminergic activity is interpreted as an *exploration bonus* that motivates animals to seek novel stimuli and situations for future reward^25^. In this context, our data suggest that VTA activation in response to visual inputs representing higher mobility can be a motivational driving force of environmental exploration, and therefore that speeding propensity might be a side effect of such exploratory motivation.

Speed-related activation changes in the VTA did not differ between participants with no interest and participants with high interest in sports cars. Although there were significant between-group differences in self-reported driving speeds, those metrics may not have reflected the actual speeding propensity in real traffic. In fact, the number of speeding offenders was similar between the two groups. In addition, although risky driving tendencies, including speeding violations, have been reported to be associated with sensation-seeking personality traits^26^, we found no significant between-group differences in SSS scores. Further validation of our hypothesis will require future studies to examine the associations between speed-related VTA activation and individual differences in speeding propensity. Group comparisons using a more reliable measure of actual speeding propensity in real traffic are desirable for future research. Combination of neuroimaging with recent technologies for recording naturalistic driving^27^ could provide a valuable methodology.

Our exploratory whole-brain analysis revealed speed-related changes in bilateral putamen activation. The putamen is also known to be a dopamine-rich brain structure that responds to reward-related sensory cues. Specifically, one study has shown putamen activation when people who received a monotonous diet before fMRI scanning imagined their favorite foods^28^. In another study, putamen activation in response to food pictures were positively correlated with the subjective feeling of appetite^29^. Furthermore, a study has shown that smoking abstinence in smokers (which creates increased craving for smoking) potentiated putamen activation in response to photographic smoking cues^30^. These findings suggest that the speed-related changes in putamen activation that we observed support our hypothesis that sensory inputs during high-speed driving modify activation within the brain-reward system.

The whole-brain analysis also revealed speed-related activation changes within the dorsal stream, as well as the ventral stream, of the visual system. In general, the dorsal visual stream is considered critical for visual-guided actions. In our experimental task, although driving-related actions (e.g., steering and using pedals) were not required, visual-guided eye movements were expected to occur because participants were asked to view the video clips as if they were the driver. Several previous studies have demonstrated that during curve negotiation, drivers keep looking at the tangent point (or its close vicinity) on the inside edges of bends^31–34^. Therefore, although eye movements were not collected during fMRI scanning in this study, it is likely that pursuit eye movements to the tangent points occurred during the task blocks, and that their velocity and frequency were greater in the fast compared with the slow condition. Therefore, speed-related activation within the dorsal visual stream may be associated with driving-related eye movements. This interpretation is consistent with the fact that greater activation with increased driving speed in the frontal eye field and the cerebellar vermis because these regions are well known to play a role in pursuit eye movements^35,36^.

Meanwhile, the exploratory PPI analysis revealed a greater functional coupling of the VTA with the medial PFC in the fast condition than in the slow condition. Functional connectivity between these two regions has been reported in a previous resting state fMRI study in healthy individuals, using the VTA as a seed region^37^. Furthermore, according to a meta-analysis of neuroimaging studies regarding positive aesthetic appraisal^38^, medial PFC contains a mosaic of small regions that are sensitive to positive valence of sensory inputs from different modalities. More specific to the vision, the medial PFC is known to be highly active when we look at beautiful paintings^39^, abstract figures^40^, and mathematical equations^41^. These lines of evidence lead us to speculate that speed-related functional coupling between the VTA and the medial PFC is related to a favorable feeling towards the visual input generated by high-speed driving. However, because participants in the current study were not required to judge the favorableness of visual stimuli, further studies are needed to assess this possibility.

Interestingly, the PPI analysis also identified a significant group difference in speed-related functional connectivity between the VTA and the right IPS. To the best of our knowledge, no study has reported contextual modulation of functional coupling between these regions. The IPS is known to play a crucial role in attentional control^42,43^. Thus, one interpretation of the result is that greater attention to visual inputs during high-speed driving in individuals with a greater propensity for speeding positively modulates the functional coupling between the VTA and the right IPS. This explanation seems to be aligned with the theoretical framework of craving (called attentional bias), in which reward-related sensory cues tend to grab the attention of people who have strong cravings^44^. Nonetheless, this is merely a reverse inference based on a functional role of the IPS and therefore needs to be clarified.

This study involved several limitations that should be considered. First, pursuit eye movements during curve negotiation are a potential confounding factor in our measure of speed-related VTA activation although previous neuroimaging studies of pursuit eye movements have not shown the involvement of the VTA^35,36^. Second, our study focused only on the visual aspects of sensory inputs during high-speed driving. Vestibular inputs such as linear acceleration and centrifugal force are also important sensory cues for high-speed driving, but were not taken into account in the present experiment. Even regarding visual inputs, the visual angle of stimulus movie clips employed in the present experiment was too small to reproduce the actual optical flow observed by drivers in real traffic. Thus, it would be valuable for future research to utilise more realistic sensory inputs during high-speed driving in an MRI environment.

## Methods

### Participants

Thirty-four healthy young adults who drove on a daily basis (with at least 2 years of experience as a licensed driver; annual mileage ≥5000 km) participated in this study. An additional inclusion criterion was based on interest in sports cars. According to a self-rating procedure using a four-point scale on interest in sports cars (1: no interest, 2: a little interest; 3: some interest; and 4: high interest), half of the participants (13 male and 4 female; age range = 24–29 years) were highly interested in sports cars (i.e., a rating of 4); the other half (12 male and 5 female; age range = 20–28 years) had no interest (i.e., a rating of 1). All participants were free from any neurological or psychiatric disorders, had normal or corrected-to-normal vision, and were right-handed according to the Edinburgh Handedness Inventory^45^.

Each participant provided written informed consent to participate in the study. The experimental protocols were approved by the ethical committees of Toyota Central R&D Laboratories, Inc and the National Institute of Physiological Sciences, and were conducted according to the principles of the Declaration of Helsinki.

### Group characteristics

To assess between-group differences in speeding propensity, we asked participants to answer two questions regarding driving speed in expressways: (1) participants’ usual speed in empty expressways with a speed limit of 100 km/h; and (2) participants’ desired speed in empty expressways with no speed limit. We also asked participants to report the number of speeding tickets they had ever received, and to complete a Japanese version^46^ of Zuckerman’s SSS questionnaire^47^ for the assessment of general propensities for stimulating and arousing experiences. The two types of driving speed and the total SSS scores were compared between groups using a t-test with a significance level of *P* < 0.05. Meanwhile, between-group differences in the proportion of speeding offenders were examined using a chi-square test with a significance level of *P* < 0.05.

### Stimuli

**Figure 6:**
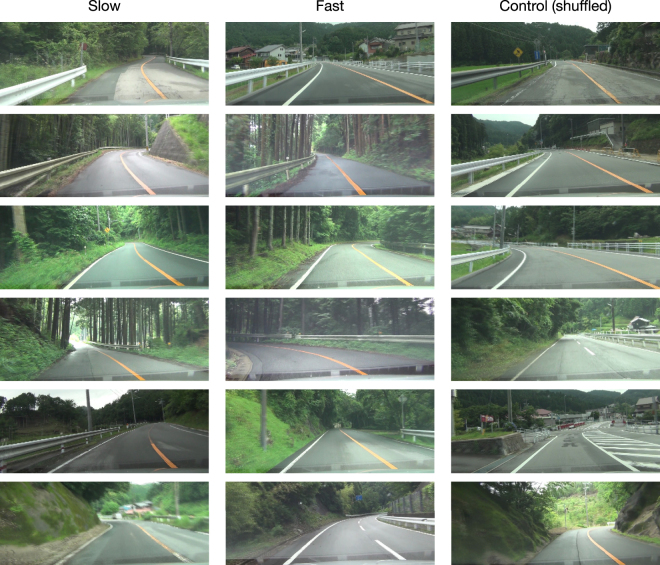
Initial frames of stimulus movie clips. Each movie clip contained 900 frames of traffic scenes on Japanese rural roads with a posted speed limit of 40 km/h, a frame rate of 60 fps, and a size of 640 × 256 pixels (width × height). Traffic scenes were recorded using a high-definition digital camera installed in a car traveling at approximately 30 km/h. In our experiments, one third of clips preserved the original speed as recorded (slow condition; left column), the other third quadrupled the speed by decimating frames (fast condition; middle column), and the remaining third consisted of randomly selected frames (control condition; right column).

Video clips of real traffic scenes were used as visual stimuli (Fig. 6). Traffic scenes were recorded with a high-definition digital camera HDR-CX270V (Sony Corp, Tokyo, Japan) installed in the dashboard of a car traveling at approximately 30 km/h on Japanese rural roads with a posted speed limit of 40 km/h. From the recorded traffic scenes, 18 scenes of 15-s duration in which no other road users were present were arbitrarily selected. Six of the scenes preserved the original speed as recorded for the slow condition (vehicle speed: M = 31, SD = 1 km/h); the other six involved quadruple speed for the fast condition (vehicle speed: M = 124, SD = 4 km/h), i.e., frames were decimated by a factor of four; and the remaining six of which consisted of randomly selected frames for the control condition. All video clips were saved at a frame rate of 60 frames per second (fps) and a size of 640 × 256 pixels (width × height; Supplementary Movies: S1, S2, and S3).

### MRI data acquisition

MRI data acquisition was performed using a 3-Tesla Siemens Verio scanner with a 32-channel head coil (Siemens, Erlangen, Germany). For functional imaging, echo planar imaging (EPI) images were acquired using a T2*-weighted gradient echo sequence with a repetition time (TR) of 3,000 ms, an echo time (TE) of 30 ms, a flip angle (FA) of 80°, a field of view (FOV) of 192 × 192 mm and a matrix size of 64 × 64. Each EPI image comprised 42 axial slices of 3-mm thickness with a 17% slice gap, which covered the entire brain. During each run, 68 images were collected, and the first three images were discarded to avoid the T1 saturation effect. In addition, a high-resolution structural brain image was acquired using a three-dimensional T1-weighted MP-RAGE sequence with a TR of 1,800 ms, a TE of 2.97 ms, an FA of 9°, an FOV of 250 × 250, a matrix size of 256 × 256, and 192 axial slices of 1-mm slice thickness.

### Procedure

All stimuli were controlled with Presentation (Neurobehavioral Systems, San Francisco, CA, USA) software, and displayed on a back-projection screen on a platform placed on the scanner bed. Stimuli on the screen were viewed through a mirror attached to the head coil. Under these viewing conditions, stimulus video clips subtended approximately 55° in horizontal and 23° in vertical visual angle.

Three fMRI scanning runs were conducted for each participant. In each run, six video clips were presented in a block design with an inter-block interval of 15 s. During intervals, a white cross was presented on a homogeneous grey background. Participants were instructed to view the video clips as if they were a driver during the task blocks, and to fixate on the white cross during the intervals. Each of the 18 video clips appeared once in one of the three runs; and the three stimulus conditions (control, slow and fast) were intermixed for each run, with the constraint that each stimulus condition appeared with a constant interval (75 s).

### fMRI data analysis

For each participant, the acquired MRI images were preprocessed using SPM12 (www.fil.ion.ucl.ac.uk/spm/). First, slice timing correction was performed, and all EPI volumes were spatially realigned for motion correction and coregistered to the participant’s T1-weighted structural image. Second, the structural image was segmented into grey matter, white matter and cerebrospinal fluid and normalized to Montreal Neurological Institute (MNI) space, using the CAT12 toolbox (dbm.neuro.uni-jena.de/cat/). Third, using the deformation field obtained through the normalization process, the coregistered EPI volumes were normalized to MNI space and resliced to 2-mm isotropic voxels. Finally, the normalised EPI volumes were spatially smoothed with an isotropic 8 mm full-width-at-half-maximum Gaussian kernel.

The preprocessed volumes were entered into a voxel-wise general linear model (GLM) to identify task-related activation for each participant. The GLM included a separate regressor for each stimulus condition (slow, fast, and control). Each regressor was generated by convolving a canonical hemodynamic response function into a boxcar function representing stimulus presentation. A temporal high-pass filter with a cutoff of 128 s was also incorporated into the GLM for baseline correction. Contrast images for the slow and fast conditions were calculated against the shuffle condition as a control.

Using the contrast images obtained from the GLM analysis, we conducted ROI analysis to validate our hypothesis that sensory inputs during high-speed driving would activate the brain reward system. To this end, we selected the VTA and the left and right NAc as mesolimbic dopaminergic ROIs. The VTA ROI was determined in accordance with the probabilistic atlas of the midbrain^48^ with a threshold of 0.7 (Fig. 2A). The bilateral NAc ROIs were determined using the Harvard-Oxford subcortical atlas with a threshold of 0.7 (Fig. 3A). Activation strength was calculated as the mean contrast estimate across voxels within each ROI. A two-by-two ANOVA with stimulus condition (slow *vs*. fast) as a within-subject factor and group (no *vs*. high interest in sports cars) as a between-subject factor was then conducted on extracted contrast estimates with a significance level of *P* < 0.05, using SPSS ver. 23 (IBM Corp., Armonk, NY, USA) software.

To explore additional brain regions associated with high-speed driving, we performed a whole-brain voxel-wise ANOVA with the same two-by-two design as the ROI analysis. We also tested whether speed-related activation changes correlated with the general propensities for stimulating and arousing experiences using individual contrast images (fast *vs*. slow) and SSS scores. In this regard, we set the level of statistical significance at family-wise error (FWE) corrected *P* < 0.05 at the cluster level with a height-threshold of *P* < 0.001 at the voxel level.

We also conducted an exploratory PPI analysis using the CONN functional connectivity toolbox (www.nitrc.org/projects/conn) to examine speed-related functional connectivity of mesolimbic dopaminergic regions with the rest of the brain. Seed regions were identical to those in the abovementioned ROI activation analysis (i.e., VTA ROI and left and right NAc ROIs). We used the default processing pipeline as implemented in the CONN toolbox. We applied the anatomical component correction method (aCompCor)^49^ to regress out physiological noise and also used the ART toolbox (www.nitrc.org/projects/artifact_detect/) to remove motion-related artefacts. As a result, we had to exclude one participant (female, age 25 years, no interest in sports cars) from all analyses because we detected excessive outlier volumes (>20%). We set the level of statistical significance at FWE corrected *P* < 0.05 at the cluster level with a height-threshold of *P* < 0.001 at the voxel level.

### Data availability statement

The datasets generated during and/or analysed during the current study are available from the corresponding author on reasonable request.

## Electronic supplementary material

Supplementary movie S1

Supplementary movie S2

Supplementary movie S3

## References

[1] Hagman O (2010). Driving pleasure: a key concept in Swedish car culture. Mobilities.

[2] Beeli G, Koeneke S, Gasser K, Jancke L (2008). Brain stimulation modulates driving behavior. Behav. Brain Funct..

[3] Erk S, Spitzer M, Wunderlich AP, Galley L, Walter H (2002). Cultural objects modulate reward circuitry. Neuroreport.

[4] Bernardi G (2014). It’s not all in your car: functional and structural correlates of exceptional driving skills in professional racers. Front. Hum. Neurosci..

[5] Calhoun VD, Pekar JJ, Pearlson GD (2004). Alcohol intoxication effects on simulated driving: exploring alcohol-dose effects on brain activation using functional MRI. Neuropsychopharmacology.

[6] Horikawa E (2005). The neural correlates of driving performance identified using positron emission tomography. Brain Cogn..

[7] Jeong M (2006). Functional brain mapping of actual car-driving using [18F]FDG-PET. Ann. Nucl. Med..

[8] Mader M (2009). Simulated car driving in fMRI–Cerebral activation patterns driving an unfamiliar and a familiar route. Neurosci. Lett..

[9] Maguire EA (2003). Navigation expertise and the human hippocampus: a structural brain imaging analysis. Hippocampus.

[10] Sakai H, Ando T, Sadato N, Uchiyama Y (2017). Greater cerebellar gray matter volume in car drivers: an exploratory voxel-based morphometry study. Sci. Rep..

[11] Sakai H, Uchiyama Y, Tanaka S, Sugawara SK, Sadato N (2014). Prefrontal transcranial direct current stimulation improves fundamental vehicle control abilities. Behav. Brain Res..

[12] Spiers HJ, Maguire EA (2007). Neural substrates of driving behaviour. Neuroimage.

[13] Uchiyama Y, Ebe K, Kozato A, Okada T, Sadato N (2003). The neural substrates of driving at a safe distance: a functional MRI study. Neurosci. Lett..

[14] Uchiyama Y (2012). Suppression of brain activity related to a car-following task with an auditory task: An fMRI study. Transp. Res. Part F Traffic Psychol. Behav..

[15] Walter H (2001). The neural correlates of driving. Neuroreport.

[16] Filbey FM (2008). Exposure to the taste of alcohol elicits activation of the mesocorticolimbic neurocircuitry. Neuropsychopharmacology.

[17] Bragulat V (2010). Food-related odor probes of brain reward circuits during hunger: a pilot FMRI study. Obesity.

[18] Due DL, Huettel SA, Hall WG, Rubin DC (2002). Activation in mesolimbic and visuospatial neural circuits elicited by smoking cues: evidence from functional magnetic resonance imaging. Am. J. Psychiatry.

[19] Acevedo BP, Aron A, Fisher HE, Brown LL (2012). Neural correlates of long-term intense romantic love. Soc. Cogn. Affect. Neurosci..

[20] Aron A (2005). Reward, motivation, and emotion systems associated with early-stage intense romantic love. J. Neurophysiol..

[21] Beaver JD (2006). Individual differences in reward drive predict neural responses to images of food. J. Neurosci..

[22] Stoeckel LE (2008). Widespread reward-system activation in obese women in response to pictures of high-calorie foods. Neuroimage.

[23] Blatter K, Schultz W (2006). Rewarding properties of visual stimuli. Exp. Brain Res..

[24] Krebs RM, Heipertz D, Schuetze H, Duzel E (2011). Novelty increases the mesolimbic functional connectivity of the substantia nigra/ventral tegmental area (SN/VTA) during reward anticipation: Evidence from high-resolution fMRI. Neuroimage.

[25] Kakade S, Dayan P (2002). Dopamine: generalization and bonuses. Neural Netw..

[26] Jonah BA (1997). Sensation seeking and risky driving: a review and synthesis of the literature. Accid. Anal. Prev..

[27] Campbell KL (2012). The SHRP 2 naturalistic driving study. Transp. Res. News.

[28] Pelchat ML, Johnson A, Chan R, Valdez J, Ragland JD (2004). Images of desire: food-craving activation during fMRI. Neuroimage.

[29] Porubská K, Veit R, Preissl H, Fritsche A, Birbaumer N (2006). Subjective feeling of appetite modulates brain activity: an fMRI study. Neuroimage.

[30] McClernon FJ, Kozink RV, Lutz AM, Rose JE (2009). 24-h smoking abstinence potentiates fMRI-BOLD activation to smoking cues in cerebral cortex and dorsal striatum. Psychopharmacology.

[31] Kandil FI, Rotter A, Lappe M (2009). Driving is smoother and more stable when using the tangent point. J. Vis..

[32] Land MF, Lee DN (1994). Where we look when we steer. Nature.

[33] Land MF, Tatler BW (2001). Steering with the head. the visual strategy of a racing driver. Curr. Biol..

[34] Mars F, Navarro J (2012). Where we look when we drive with or without active steering wheel control. PLoS One.

[35] Krauzlis RJ (2004). Recasting the smooth pursuit eye movement system. J. Neurophysiol..

[36] Tanabe J, Tregellas J, Miller D, Ross RG, Freedman R (2002). Brain activation during smooth-pursuit eye movements. Neuroimage.

[37] Gu H (2010). Mesocorticolimbic circuits are impaired in chronic cocaine users as demonstrated by resting-state functional connectivity. Neuroimage.

[38] Brown S, Gao X, Tisdelle L, Eickhoff SB, Liotti M (2011). Naturalizing aesthetics: brain areas for aesthetic appraisal across sensory modalities. Neuroimage.

[39] Ishizu T, Zeki S (2013). The brain’s specialized systems for aesthetic and perceptual judgment. Eur. J. Neurosci..

[40] Jacobsen T, Schubotz RI, Höfel L, Cramon DYV (2006). Brain correlates of aesthetic judgment of beauty. Neuroimage.

[41] Zeki S, Romaya JP, Benincasa DMT, Atiyah MF (2014). The experience of mathematical beauty and its neural correlates. Front Hum Neurosci.

[42] Corbetta M, Patel G, Shulman GL (2008). The reorienting system of the human brain: from environment to theory of mind. Neuron.

[43] Corbetta M, Shulman GL (2002). Control of goal-directed and stimulus-driven attention in the brain. Nat. Rev. Neurosci..

[44] Field M, Munafò MR, Franken IHA (2009). A meta-analytic investigation of the relationship between attentional bias and subjective craving in substance abuse. Psychol. Bull..

[45] Oldfield RC (1971). The assessment and analysis of handedness: the Edinburgh inventory. Neuropsychologia.

[46] Terasaki M, Shiomi K, Kishimoto Y, Hiraoka K (1987). A Japanese version of sensation-seeking scale. Jpn. J. Psychol..

[47] Zuckerman M, Kolin EA, Price L, Zoob I (1964). Development of a sensation-seeking scale. J. Consult. Psychol..

[48] Murty VP (2014). Resting state networks distinguish human ventral tegmental area from substantia nigra. Neuroimage.

[49] Behzadi Y, Restom K, Liau J, Liu TT (2007). A component based noise correction method (CompCor) for BOLD and perfusion based fMRI. Neuroimage.

